# Erdheim-Chester disease as complex clinical presentation and diagnosis: A case report and concise review of literature

**DOI:** 10.1097/MD.0000000000037870

**Published:** 2024-04-26

**Authors:** Carola M. Gagliardo, Antonina Giammanco, Augusto Vaglio, Francesco Pegoraro, Angelo B. Cefalù, Maurizio Averna, Davide Noto

**Affiliations:** a Department of Health Promotion, Maternal and Child Health, Internal and Specialized Medicine of Excellence “G. D. Alessandro” (PROMISE), University of Palermo, Palermo, Italy; b Nephrology and Dialysis Unit, Meyer Children’s Hospital IRCCS, Florence, Italy; c Department of Biomedical, Experimental and Clinical Sciences “Mario Serio,” University of Florence, Florence, Italy; d Department of Health Science, University of Florence, Florence, Italy; e Pediatric Hematology and Oncology Unit, Meyer Children’s University Hospital IRCCS, Florence, Italy.

**Keywords:** *BRAFV600E* mutation, case report, Erdheim-Chester disease (ECD), histiocytosis, non-Langerhans cell histiocytosis

## Abstract

**Rationale:**

Erdheim-Chester disease (ECD) is a rare multisystemic disease characterized by the infiltration of multiple organs by foamy CD68 + CD1a-histiocytes. The genetic background consists of gain-of-function somatic mutations in the mitogen-activated protein kinase pathway. The purpose of the present paper is to make a contribution to the scientific literature on ECD by reporting our experience with a complex clinical case report, along with a concise review of the literature. We discussed the unusual clinical presentation, the complex diagnostic process and the comparison with other published cases.

**Patient concerns:**

A 70-year-old man presented with arthralgia due to multiple bone areas of sclerosis, first diagnosed with metastases of a prostatic neoplasm. Sequential thorax-abdomen, femoral and homer contrast-enhanced computed tomography (CT) showed pericardial effusion, pulmonary fibrosis, and perirenal fibrous tissue as “hairy kidneys.” He underwent. Three bone biopsies were unsuccessful to reach diagnosis.

**Diagnoses:**

A xanthelasma biopsy showed histopathological signs compatible with ECD; genetic analysis showed the mutation BRAFV600E.

**Interventions:**

The patient underwent targeted therapy with vemurafenib (BRAF-inhibitor), discontinued 2 weeks later due to the onset of a diffuse erythematous papular rash on the trunk and limbs.

**Outcomes:**

At the 1-year follow-up, there was only progression of chronic kidney disease (CKD).

**Lessons:**

The present case report describes how ECD diagnosis could represent a challenge for clinicians, owing to its heterogeneous clinical presentation. Early diagnosis followed by prompt therapy is essential for modifying the natural history of the disease.

## 1. Introduction

Erdheim-Chester disease (ECD) is a rare multisystemic disease characterized by the infiltration of multiple organs by foamy CD68 + CD1a- histiocytes (resident mononucleate macrophages) and “Touton” cells (multinucleated giant cells), which are typical of lesions with high lipid content, such as fat necrosis, xanthomas, and xanthelasmas. ECD is classified within the group of “L” histiocytosis, together with Langerhans cell histiocytosis, indeterminate cell histiocytosis and mixed Langerhans cell histiocytosis and EDC.^[1]^

Histiocytoses are rare (annual incidence of less than 5 cases per million inhabitants) and heterogeneous disorders characterized by the accumulation of macrophages, dendritic cells, or monocyte-derived cells in various tissues and by different clinical presentations, ranging from isolated cutaneous manifestations to life-threatening neoplastic conditions.^[1]^

ECD spreads multisystemically in most cases, affecting the cardiovascular, endocrine, and central nervous systems, and causes long bone osteosclerosis, retroperitoneum, lung and skin fibrosis, facial sinus, and bone remodeling.^[2]^ Skeletal involvement occurs in 95% of ECD patients with bilateral symmetric cortical long bone osteosclerosis at the meta diaphysis.^[1]^ Involvement of the hypothalamus-pituitary axis is common, and central diabetes insipidus (CDI) represents the first clinical manifestation of ECD in 25% to 48% of cases.^[3]^ ECD histiocytes harbor somatic mutations in genes of the mitogen-activated protein kinase pathway, which determine mitogen signaling during cell cycle progression and induce cell proliferation. Proinflammatory signaling also plays a role in disease progression.^[4]^ The resulting multiorgan damage mimics that of neoplastic and systemic immune-mediated diseases.^[5]^ The ECD genetic background consists of somatic gain-of-function mutations in approximately 80% of patients, and the activating mutation in BRAF c.1799T > A, p.Val600Glu (BRAFV600E) is found in 57% to 70% of cases, followed by MAP2K1 mutation, accounting for another 20% of cases.^[1,5]^ Genetic characterization allows the use of specific targeted therapies. The diagnosis of ECD is frequently delayed owing to its heterogeneous clinical presentation, which in most cases is challenging for clinicians.^[6]^

We present the case of a 70-year-old man who consulted physicians for leg bone pain caused by multiple bone areas of osteosclerosis and was first diagnosed with metastases from a prostatic neoplasm. ECD was diagnosed 1 year later.

The patient presented almost all possible clinical manifestations of the disease but the diagnosis was nevertheless obtained after more than a year of investigation. We believe that the description of the entire diagnostic process, the unusual clinical presentation and the enrichment with the literature review may be of considerable interest to the scientific community.

## 2. Methods

De-identified data on a 60-year-old patient were collected during medical examinations at our center. The patient provided his written informed consent. Approval by ethical committee was obtained to practice off-label therapy with vemurafenib, conversely, the approval was not required to publish the case report, according to the Italian “Superior Institute of Health,” as the paper does not describe any clinical, observational, epidemiological study protocols.

The literature search was carried out on PubMed from January 2023 to August 2023, examining case reports and review articles on EDC. A total of 66 studies were identified. Articles were then filtered according to the following criteria: English language and full-text availability, and articles describing the patients’ clinical presentation, diagnostic workup, or proposed therapy. As this study was not designed as a Systematic Review of the literature, its methodological quality was not assessed.

## 3. Case presentation

A 70-year-old male was admitted to the emergency department in July 2020 because of an abdominal pain. His medical history included CDI treated with desmopressin, arterial hypertension treated with ramipril, benign prostatic hypertrophy, depression, and memory disorders.

- Abdominal computed tomography (CT) did not reveal pathological findings explaining the abdominal pain; conversely, it showed osteosclerotic lesions of the pelvis and proximal portion of the femurs, prostatic hypertrophy with uneven density, and bilateral thickening of the perirenal fascia.
- Thoracic CT revealed massive pericardial effusion (maximum thickness, 5 cm) that required prompt pericardiocentesis.
- Further contrast-enhanced CT and magnetic resonance imaging (MRI) of the abdomen and pelvis (September 2020) confirmed an inhomogeneous prostatic structure with nodularity; bilateral osteosclerosis of the femur, iliac bone, and pelvic skeletal segments was also noticed. The patient also complained of diffuse bone pain.
- Radiographs of humeri, femurs, legs, and pelvis documented areas of hyperdiaphany surrounded by osteosclerosis. The periosteum was thickened.

Prostate cancer with bone metastasis was suspected based on uneven prostatic density, despite prostatic soluble antigen (PSA) values within the normal range.

- Technetium-99m methyl diphosphonate bone scintigraphy showed intense bilateral uptake of the right humeral diaphysis, left humeral head, femoral and tibial diaphysis, and iliac crests.
- 18-f fluorodeoxyglucose whole-body positron-emission tomography (18-FDG PET) confirmed the same bone areas.
- Routine laboratory evaluation showed mild hypochromic normocytic anemia, a slight elevation in erythrocyte sedimentation rate (ESR) and C-reactive protein levels, and free and total PSA levels within the normal range. Slight increases in osteocalcin, serum beta2-microglobulin, and hydroxyprolinuria were also detected (Table 1).

**Table 1:** List of biochemical values of the patient.

Biochemical values	Reference values	July 2020	May 2021	December 2021	September 2022
WBC	4–11 × 10^3/µL	7.75	5.7	7.59	5.2
Neutrophils	40–74%	65.9	57.9	59	58.7
Lymphocytes	20–48%	22.5	25.3	23.1	29.5
Monocites	3–11%	9.8	11.8	14.2	9.7
Eosinophils	0–8%	1.4	4.6	3.2	1.7
Basophils	0–1.5%	0.4	0.4	0.5	0.4
RBC	4.2-5.5 × 10^6/µL	4.05	4.38	4.47	3.52
HB	12–18 gr/dL	**10.3**	**10.5**	**11.1**	**9.1**
Hct	37–52%	31.7	34.5	36.3	28
MCV	80–99 fL	78.3	78.8	81.2	79.5
PLT	150–450 × 10^3/µL	292	252	244	224
B2-microglobulin	0.8–2.2 mg/L	**3.22**	**3.36**	NA	NA
Osteocalcine	14–46 mcg/L	**52**	**56.8**	NA	NA
Hydroxiprolinuria	5–17 mg/24h	**54**	NA	NA	NA
Uremia	10–71 mg/dL	25	34	18	32
Creatinine	0.67–1.17 mg/dL	0.97	1.03	**1.15**	**1.46**
EGFR	90–120 mL/min	**62**	**58**	**51**	**38**
PTH	15–65 ng/L	NA	**68**	**71.1**	**83**
D-vitamine	> 30 mcg/L	NA	18	11.3	9
Ferritin	30–400 ng/mL	214	NA	199	285
Transferrin	200–360 ng/mL	**181**	NA	217	132
Iron	33–193 mcg/dL	**20**	NA	37	**28**
CRP	0–5 mg/L	**21.2**	**19.03**	**31.2**	**36.6**
INR	0.8–1.2	1.3	1.07	1.05	1.03
aPTT	24–36 sec	28	31	30	29
Fibrinogen	150–450 mg/dL	**637**	**538**	**521**	441

The table lists the main biochemical values of the patient during the follow-up. Altered values have been highlighted in bold type.

aPTT = activated partial thromboplastin time, CRP = c-reactive protein, EGFR = estimated glomerular filtration rate, HB = hemoglobin, Hct = hematocrit, INR = International Normalized Ratio, MCV = Mean Cell Volume, NA = not available, PLT = platelets, PTH = parathormone, RBC = Red blood cells.

Three bone biopsies showed nondiagnostic findings: intertrabecular fibrosis and edema, chronic inflammatory infiltrate, and diffusely coated cellular elements.

The patient visited our department in March 2021 with a suspected metabolic osteopathy. General physical examination showed xanthelasmas in the inner margin of the left eyelid.

**Figure 1.**
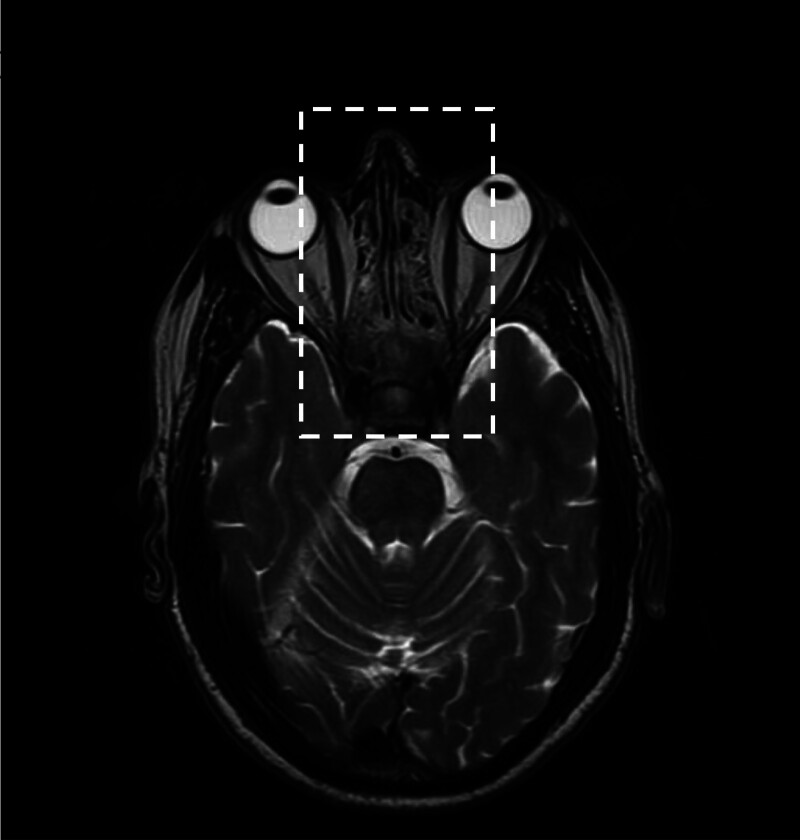
Brain MRI showing diffuse remodeling of the clivus and the ethmoid bone. The figure is captured by a brain MRI in T2 weighted-sequence. The dashed box frames diffuse remodeling of the clivus and the ethmoid bone. MRI = magnetic resonance imaging.

**Figure 2.**
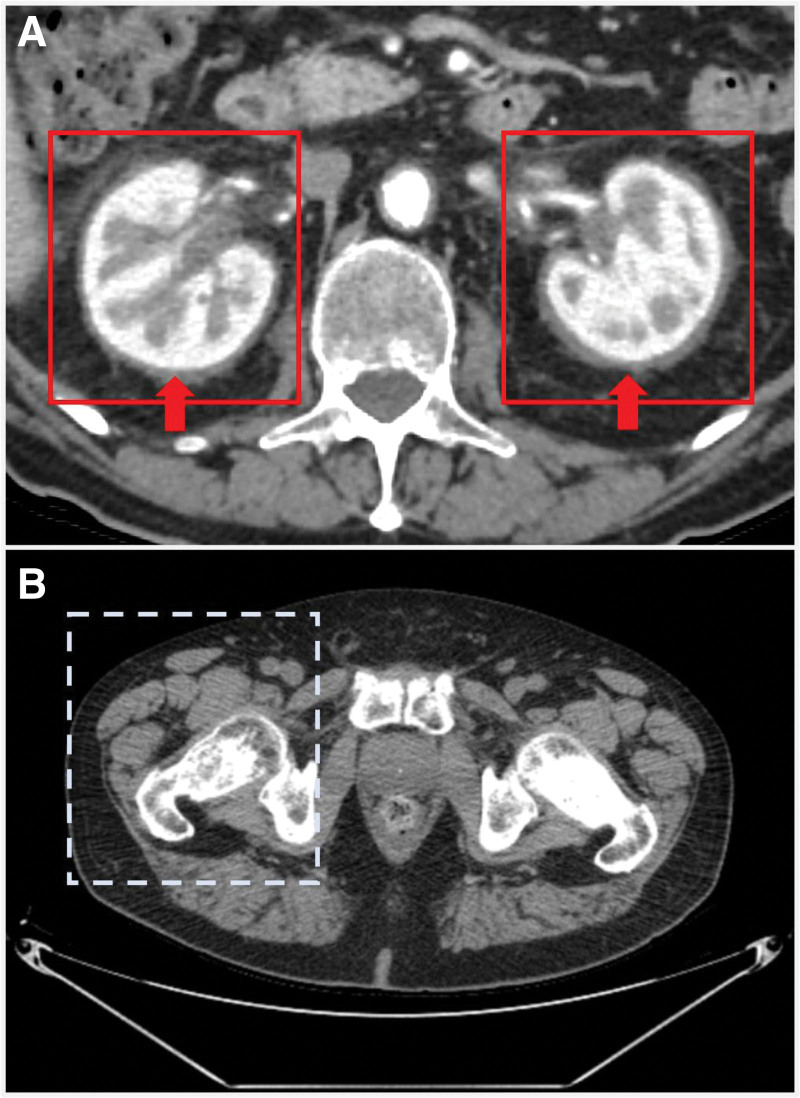
Typical findings of ECD at the CT abdomen with contrast medium. The figure illustrates typical findings of ECD at the CT abdomen without and with contrast medium. (A) Arterial phase of the CT abdomen with contrast medium. The 2 red box frame the “hairy kidneys”: the 2 red arrows indicate the perirenal fibrosis that confers the spiculated appearance to the kidneys. (B) The figure illustrates the osteosclerosis of the femoral diaphysis, the dashed box frames the osteosclerosis more evident on the right femoral bone. CT = computed tomography, ECD = Erdheim-Chester disease.

- Brain MRI showed diffuse osteostructural remodeling of the clivus, ethmoid bone, and maxillary sinuses, but did not document parenchymal lesions in the hypophysis compatible with diabetes insipidus (Fig. 1).
- A new thorax-abdomen and femoral and homer contrast-enhanced CT showed a reduction in the pericardial effusion (2.4 vs 5 cm), pulmonary apical fibrosis, and perirenal fibrous tissue along the anterior and posterior para-renal fascia, with spiculated appearance, as from “hairy kidneys,” also extending to the proximal ureteral tract with a partial compressive effect (Fig. 2A).
- A new CT scan also revealed long bone osteosclerosis of the pelvis and metadiaphyseal segments of the femur and homer bilaterally (Fig. 2B).

## 4. Clinical outcomes

A clinical diagnosis of ECD was suspected based on an objective examination of xanthelasmas, together with tomographic findings of retroperitoneal fibrosis (RPF), neurological involvement, diabetes insipidus, and bone lesions. Skin biopsy of xanthelasmas was performed in May 2021, documenting infiltration of foamy histiocytes, expressing CD68 (+), Cd1a (−), BRAFV600E (−) at the immunohistochemical analysis.^[7]^ Genetic evaluation through Polymerase Chain Reaction (PCR) amplification was performed despite the negative results of immunohistochemical analysis, and the BRAFV600E mutation was identified.

Targeted therapy with vemurafenib (a BRAF-inhibitor) was administered after agreement with the hospital ethics committee. Two weeks later, the drug was discontinued owing to the appearance of a diffuse erythematous papular rash on the trunk and limbs according to the drug data sheet.^[8]^ The rash regressed with steroid and antihistamine therapy within a few days, but the patient declined to take the drug again.

At the last follow-up in September 2022, there was no evidence of radiological progression of bone lesions. Laboratory findings showed a progressive increase in C-reactive protein levels and persistence of microcytic hypochromic anemia, probably due to chronic inflammation. A progression of the chronic kidney disease (CKD) was evidenced by the increased serum creatinine (1.46 vs 0,93 mg/dL in 2020), with consequent reduction of the estimated glomerular filtration (38 vs 62 mL/min in 2020) and by the worsening of secondary hyperparathyroidism (see Table 1).

## 5. Discussion

The case presented here fully describes the multiorgan manifestations of ECD, including osteosclerotic bone lesions, diabetes insipidus, pericardial effusion, and perirenal fibrosis. We consider this clinical case to be of interest also due to its unusual onset and the difficulty in reaching an early diagnosis.

The multiple clinical facets of the disease presented by the patient offered an important cue to enrich the current literature with a concise review of the clinical characteristics of ECD, as a completion of this complex clinical case description.

### 5.1. Skeletal involvement

Skeletal involvement is the most frequent manifestation of ECD, affecting–80% to 95% of patients. Bilateral long bone osteoclastogenesis at the meta diaphysis is considered almost exclusive to ECD.^[9]^ Leg bone pain is usually a hallmark of the disease and is the first clinical manifestation that deserves steroidal treatment. Bone lesions can be detected using conventional radiological imaging (X-rays, CT scans, MRI, bone scintigraphy, or by PET emission tomography). Technetium-99m methyl diphosphonate bone scintigraphy is useful in differentiating ECD from other sclerotic diseases. Alternative diagnoses include osteomyelitis, Paget disease, Graves’ disease, lymphoma, sarcoidosis, metastases, and lipid storage diseases. An 18-FDG PET scan is informative for the assessment of ECD activity, showing 18-FDG-avidity uptake in the long bone metadiaphysis. 18-FDG PET is particularly useful for follow-up and therapeutic responses.^[10]^

Osteolytic lesions are atypical for ECD. Conversely, these are typical findings for either LHC or a combined form of ECD/LH.^[9]^ Radiography of the skeletal segments of our patient showed areas of hyperdiaphany compatible with areas of osteolysis, surrounded by osteosclerosis. All other second- and third-level instrumental examinations indicated only osteosclerosis. Therefore, the hypothesis of a combined form of ECD/LH was not considered in our patient.

### 5.2. Retroperitoneal fibrosis

RPF occurs either as an IgG4-related (idiopathic) disease or as a feature of other disorders (drugs, infections, and neoplasms).^[11]^ IgG4-related RPF differs from ECD RPF both in CT and MRI radiological presentation: ECD-related fibrosis generally affects the perirenal area, renal pelvis, and proximal ureters; pelvic ureter and inferior vena cava are usually spared. ECD fibrosis entirely encircled the abdominal aorta. IgG4-related RPF usually develops around the anterolateral sides of the abdominal aorta but spares its posterior wall, involves the iliac arteries, and the distal part of the ureters does not obstruct the ureters but determines the medial ureteral deviation. The inferior vena cava may also be involved. At unenhanced CT scans, all forms of RPF appear as isodense masses.^[2,12]^

Histiocytic infiltration of the retroperitoneal space in ECD frequently leads to diffuse bilateral infiltrative perirenal soft tissue thickening with a spiculate pattern, defined as “hairy kidney,” which is highly suggestive of disease and has been reported in approximately 75% of ECD patients.^[13]^ In fact, 44% of patients with peri-kidney involvement develop CKD III-V stadium according to the CDK-EPI classification at the year follow-up, and conventional therapies still result in the ineffective prevention of CKD progression. Moreover, age > 50 years represents a risk factor for CKD worsening in ECD,^[14]^ and our case report testimonies a 38% reduction rate over 2 years.

### 5.3. Neurological and endocrinal manifestations

Central nervous system (CNS) disorders occur in approximately 40% of patients with ECD and increase the risk of mortality.^[1]^ Cerebellar and pyramidal syndromes are the most common neurological signs; however, seizures, headaches, cognitive impairment, cranial nerve palsy, neuropsychiatric signs, and asymptomatic lesions have also been described. Our patient showed behavioral disorders (anxious-depressive state) and intermittent loss of memory, suggesting CNS involvement, which was also confirmed by the T2-sequences on encephalic MRI showing a small vessel disease that could be responsible for the neurological features. Nevertheless, these findings are not exclusive to ECD. Intracranial arterial infiltration can be considered as another feature of ECD that leads to ischemic stroke. One-quarter of all patients develop exophthalmos due to infiltration of retro-orbital soft tissues, and this condition is often bilateral. Maxillary and sphenoid sinus infiltrations are common (47%), whereas ethmoidal and frontal sinus infiltrations (17%) are less common.^[1]^ Neuroradiological findings were categorized into 3 patterns: infiltrative pattern (44%), with widespread lesions, nodules, or intracerebral masses; meningeal pattern (37%), with thickening of the dura mater or meningioma-like tumors; and composite pattern (19%), with both infiltrative and meningeal lesions.^[15]^

CDI represents the first clinical manifestation of ECD in 25% to 48% of cases. CDI can develop several years before ECD diagnosis, and its pathogenesis seems to be related to pituitary infiltration by histiocytes.^[16]^ Other forms of pituitary dysfunction include hyperprolactinemia (44.1%), somatotropic (78.6%), gonadotropic (22.2%), thyrotropic (9.5%), and corticotropic (3.1%) deficiencies.^[3]^ However, brain MRI did not show pituitary infiltration in our patient despite the clinical diagnosis of CDI, and no additional hormonal deficits related to pituitary disorders were found.

### 5.4. Cardiovascular involvement

The most frequent cardiovascular sign is aortic sheathing (“coated aorta”), revealed by CT scans in 46% to 62% of cases. It is usually asymptomatic and is not associated with dilation, dissection, or aneurysm.^[3]^ Pericardial disease is a typical finding in ECD (29%). It may present as pericarditis, pericardial effusion, or cardiac tamponade. Massive effusions are rare.^[17]^ In our patient, massive pericardial effusion was the earliest sign that raised the suspicion of ECD. Infiltration of coronary arteries can occur in 23% of patients and can lead to ischemic cardiomyopathy.^[2]^

The right atrium pseudotumor is another typical finding on cardiac MRI, occurring in 40% of ECD patients, and is frequently associated with the *BRAFV600E* mutation.^[17]^

### 5.5. Cutaneous manifestations

Xanthelasma-like lesions occur in 25% to 30% of patients. Xanthelasma-like lesions involving the reticular dermis are composed of multinucleated Touton cells and show less extensive fibrosis than classic xanthelasma palpebrarum.^[18]^ Other ECD cutaneous lesions include papulonodular lesions of the legs, back, and/or trunk.^[19]^

### 5.6. Pulmonary and other organ manifestations

The pleura and lung parenchyma can infiltrate in 30 to 50% of cases, leading to interstitial lung diseases. The thyroid, breast, and lymph nodes have also been reported to infiltrate.^[3]^

### 5.7. Diagnosis

The diagnosis requires both clinical and histopathological findings.^[2]^ The histopathological signs of ECD include organ infiltration by foamy or lipid-laden histiocytes, fibrosis, and Touton cells. Immunohistochemistry usually shows a characteristic staining profile of ECD cells (positive CD68, CD163, and factor XIIIa and negative CD1a and CD207), whereas S-100 positivity is rare.^[18]^ Immunohistochemical analysis of BRAFV600E staining detected mutant protein in histiocytic neoplasms in more than 50% of ECD patients. However, in some cases, immunohistochemistry does not detect mutant proteins. Therefore, it is preferable to perform genetic investigations to confirm or detect the correct mutation, as in our patient.^[1]^

### 5.8. Therapy

Current therapies are tailored to detect mutations, avoiding the administration of interferon-alpha as first-line therapy. The Food and Drug Administration approved vemurafenib as a first-line therapy for patients harboring the *BRAFV600E* mutation, whereas the European Medicines Agency did not approve off-label treatment.^[20]^ Adverse events associated with vemurafenib include articular pain, maculopapular skin rash, prolonged QT interval, and alopecia. Serious adverse events include different skin cancers (squamous cell carcinoma, melanoma or others), hyper-sensitivity skin reactions (anaphylaxis and DRESS syndrome), uveitis, hepatic and renal disorders, Stevens-Johnson syndrome and toxic epidermal necrolysis.^[6,21]^ Our patient showed a maculopapular skin rash 2 weeks after therapy initiation, which regressed with steroids. Different clinical trials testing vemurafenib in melanomas showed that approximately 48% of patients manifested a mild to moderate cutaneous rash, and 5% of patients experienced a severe rash during the first weeks of administration.^[22]^ According to the datasheet, the drug should be temporarily discontinued in cases of cutaneous manifestations.^[8]^ In our case, the drug was permanently discontinued due to the patient personal choice.

The MEK inhibitor cobimetinib represents a therapeutic alternative to vemurafenib, as more than 25% of patients present with mitogen-activated protein kinase pathway activating mutations (*MAP2K1, KRAS*, and *NRAS*) other than *BRAFV600E*. This drug has been demonstrated to be safe and efficacious in disease stabilization in patients with *BRAFV600E* mutation who experienced adverse events (including DRESS syndrome) after vemurafenib treatment.^[23]^ The patient refused this therapeutic option.

### 5.9. Limits of the study

Our work describes a single case report, so data are limited even if compared with available literature. The study case refused further treatments, so we do not have information regarding long-term effects of the target therapy.

## 6. Conclusions

The present clinical case highlights the risk of ECD misdiagnosis due to its elusive presentation. Simultaneous multiorgan involvement by fibrotic damage associated with bone remodeling should soon lead to the suspicion of a single pathology to which all manifestations can be traced. Moreover, the contemporary presence of pericardial effusion, diabetes insipidus, RPF, and pain should raise the suspicion of ECD.

Knowledge of such rare clinical entities can enable clinicians to make a correct diagnosis. Early diagnosis followed by prompt therapy is essential for modifying the natural history of the disease.

**Patient perspective**: the patient “Reaching the diagnosis, and thus having excluded the possible presence of malignancy, has greatly improved my psychological state and quality of life. For a long time, I had to make several specialist visits without coming to any conclusion. Now I am in follow-up at the Rare Metabolic Diseases ambulatory of the University Hospital of Palermo and I feel well cared.”

## Author contributions

**Conceptualization:** Carola Maria Gagliardo, Antonina Giammanco, Augusto Vaglio, Davide Noto.

**Data curation:** Francesco Pegoraro.

**Investigation:** Antonina Giammanco, Augusto Vaglio, Maurizio Averna, Davide Noto.

**Supervision:** Angelo Baldassare Cefalù, Maurizio Averna.

**Writing – original draft:** Carola Maria Gagliardo.

**Writing – review & editing:** Carola Maria Gagliardo, Francesco Pegoraro, Angelo Baldassare Cefalù, Davide Noto.
